# Investigations on historical monuments’ deterioration through chemical and isotopic analyses: an Italian case study

**DOI:** 10.1007/s11356-021-15103-x

**Published:** 2021-06-29

**Authors:** Maria Ricciardi, Concetta Pironti, Oriana Motta, Rosa Fiorillo, Federica Camin, Antonio Faggiano, Antonio Proto

**Affiliations:** 1 Department of Medicine Surgery and Dentistry, University of Salerno, via S. Allende, 84081 Baronissi, SA Italy; 2 Department of Cultural Heritage, University of Salerno, via Giovanni Paolo II 132, 84084 Fisciano, SA Italy; 3 Food Quality and Nutrition Department Research and Innovation Centre, Fondazione Edmund Mach (FEM), Via E. Mach 1, 38010 San Michele all’Adige, TN Italy; 4 Center Agriculture Food Environment (C3A), University of Trento, via Mach 1, 38010 San Michele all’Adige, TN Italy; 5 Department of Chemistry and Biology, University of Salerno, via Giovanni Paolo II 132, 84084 Fisciano, SA Italy

**Keywords:** Saltpetre, Efflorescences, Cultural heritage, Fresco, Nitrogen stable isotope ratio

## Abstract

In this paper, we analysed the efflorescences present in the frescos of a monumental complex named *S. Pietro a Corte* situated in the historic centre of Salerno (Campania, Italy). The groundwater of the historic centre is fed by two important streams (the Rafastia and the Fusandola) that can be the sources of water penetration. The aims of this work are to (i) identify the stream that reaches the ancient *frigidarium* of *S. Pietro a Corte* and (ii) characterize the efflorescences on damaged frescos in terms of chemical nature and sources. In order to accomplish the first aim, the water of the Rafastia river (7 samples) and the water of the Fusandola river (7 samples) were analysed and compared with the water of a well of the Church (7 samples). The ionic chromatography measurements on the water samples allowed us to identify the Rafastia as the river that feeds the ancient *frigidarium* of *S. Pietro a Corte*. To investigate the nature and the origin of the efflorescences (our second aim), anionic chromatography analyses, X-ray diffraction measurements, and the isotopic determination of nitrogen were performed on the efflorescences (9 samples) and the salts recovered from the well (6 samples)*.* Results of these analyses show that efflorescences are mainly made of potassium nitrate with a δ^15^N value of + 9.3 ± 0.2‰. Consequently, a plausible explanation for their formation could be the permeation of sewage water on the walls of the monumental complex.

**Supplementary Information:**

The online version contains supplementary material available at 10.1007/s11356-021-15103-x.

## Introduction

### Investigations on the deterioration of cultural heritage caused by efflorescences

Over the past decades, the degradation of cultural heritage due to exposure to atmospheric and water pollution has become an increasing concern, thus highlighting the need to know the origin and the evolution of pollutants to preserve it (Alfano et al. 2009; Di Turo et al. 2016).

Several air pollutants (NO_x_, SO_x_, and CO_2_) have negative effects on cultural heritage because of their chemical reaction with raw materials, so many efforts were devoted to finding simple methods able to monitor their concentration (Cucciniello et al. 2012, 2017; Motta et al. 2014, 2018).

The formation of efflorescences is one of the most important issues concerning the preservation of cultural heritage. Efflorescence is the migration of salts to the surface of a porous material where they form a coating. The process involves three main steps: (i) the dissolution of salts in water in the internal part, (ii) the migration of water and salts to the surface, and (iii) the evaporation of water leaves a coating of salts. Consequently, they damage sculptures and frescos and are easily reformed a few months after their mechanical removal (Backbier et al. 1993; Gázquez et al. 2015; Siedel 2018; Alexandrowicz and Marszałek 2019). The knowledge of nature and the sources of efflorescences is fundamental to prevent their formation and so avoid the deterioration of cultural heritage. Several salts with high solubility can form efflorescences in walls such as chlorides, sulphates (especially of calcium and sodium), carbonates, nitrates (e.g. potassium nitrate or saltpetre), oxalates of sodium, potassium, calcium, magnesium, and ammonia (Arnold and Zehnder 1991); therefore, a chemical analysis is mandatory to assess the composition of efflorescences. Different mechanisms are involved in the formation of these salt coatings (Scrivano and Gaggero 2020), and in the case of saltpetre efflorescences, possible sources are the nitrates present in the infiltration groundwater and the atmospheric nitrogen oxides (NO_x_).

Stable isotope ratio analysis demonstrated the potential to differentiate between nitrate samples from different origins (Benson et al. 2006), e.g. in the investigation of complex forensic cases by identifying different sources of the same type of explosive, namely, ammonium nitrate. Nitrogen stable isotope ratio expressed as δ^15^N is useful in discriminating the fount of a specific nitrogen compound such as in sediments, where it indicates the contribution of human waste to total nitrogen (Bedard-Haughn et al. 2003), and investigating the nitrogen cycle in ecosystems (Li and Wang 2008; Huber et al. 2011).

Moreover, it has been widely used to estimate the origin of nitrates (NO_3_^-^) in water (Wells and Krothe 1989; Haberhauer et al. 2002; Kellman and Hillaire-Marcel 2003; Xue et al. 2009; Fenech et al. 2012), because the nitrogen isotopic composition in NO_3_^-^ usually changes depending on its different sources (e.g. mineral fertilizers, manure, soil, and atmospheric N_2_). Some issues such as mixing of distinct sources and kinetic isotopic fractionation (e.g. denitrification) (Kendall et al. 2007) must be taken into account to achieve a proper identification of nitrate’s origin. In some cases, a combination of nitrogen and oxygen isotope values provides better discrimination of nitrate sources (Benson et al. 2009; Hosono et al. 2013; Zhao et al. 2019; Jia et al. 2020).

In the field of cultural heritage, stable isotope ratio was successfully utilized to discover the origin of gypsum-rich black crusts developed on granites (Rivas et al. 2014) and sulphates and nitrate efflorescences in sandstones from monuments (Schweigstillová et al. 2009; Schleicher and Recio Hernández 2010) and porous limestone sculptures (Kloppmann et al. 2014; Gázquez et al. 2015).

Consequently, the isotopic approach can be a very useful tool in combination with chemical investigations to study the deterioration of historical monuments.

### Historical, geographical, and archaeological contextualization of the case under study

**Fig. 1:**
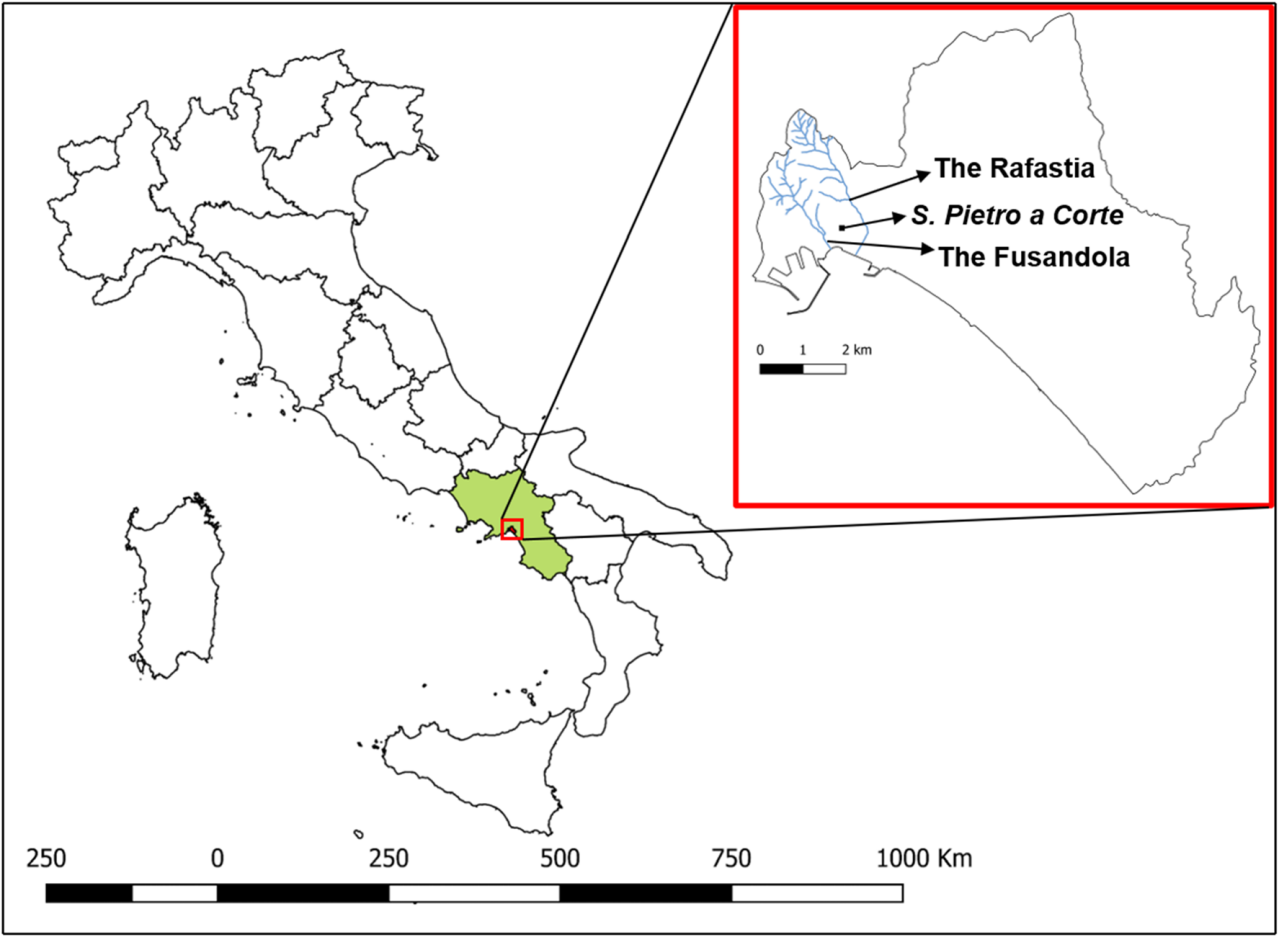
Map of Italy: the Region Campania is shown in green, while the magnification of the city of Salerno with the delimitation of Municipality in black, the course of the two streams and the position of the Monumental complex are shown in the red square

The colony of Salerno was founded by the Romans in 197 BC. During the Roman period, the city of Salerno was delimited by two small rivers named the Rafastia (Nord-West) and the Fusandola (Nord-East) (Fig. 1). Few traces of this period are now visible because many buildings were built during the Middle Ages, often on areas buried by the debris of the floods of these rivers which periodically affected the city (Amarotta 1989; Longo 2020; Fiorillo 2020).

In the eighth century AD, the Lombard prince Arechi II erected in Salerno, the capital of his kingdom, an autonomous structure intended for the function of the palatine chapel that now is the *S. Pietro a Corte* Church. In the following years, the chapel had different uses, from an ideal place for graduation ceremonies of the Salerno Medical School (the oldest Medical School of Europe) in the Middle Ages to a military storage area during the First World War (Fiorillo 2013).

Starting from the 1950s, the presence of a water well in the underside of the church has been documented by some bakers who used to take water from it, carrying out their activities in these environments. In 1970, the collapse of the floor at street level showed some frescos on the underside of the Monumental Complex of *S. Pietro a Corte* (see Figure S1-a for section and planimetry), and a few years later, in 1980, an earthquake caused the complete fall of the floor of this monumental complex.

These episodes prompted archaeologists to carry out some excavations that have brought to light two complexes (Figure S1-b) at about six meters below the current street level: one in the West Side consisting of the thermal structure of a *frigidarium* pool dating back to the first or second century AD, and another in the East (Figure S1-c”) containing a temple of Priapus, later to become a Paleochristian Church having some wall frescos (Fiorillo 2013). The other part of the thermal complex was discovered from the excavations in the *Palazzo Fruscione* that is located on the north side, in front of *S. Pietro a Corte*, separated by a path of few meters wide, now known as Vicolo Adelberga. Probably, the well discovered as the feeding of the *frigidarium* (Figure S2) was the same used by local bakers for taking water.

**Fig. 2:**
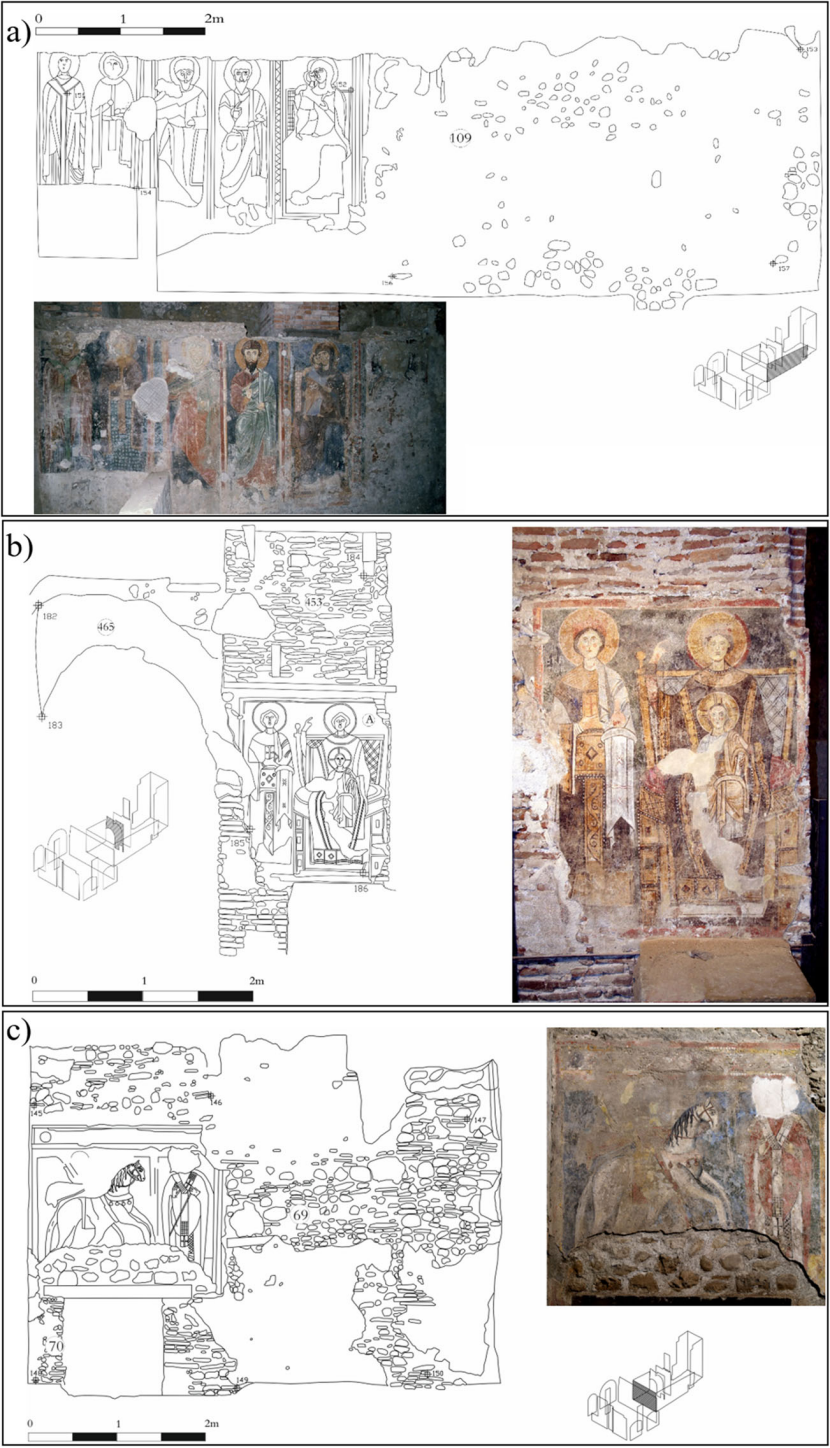
Discovered frescos with their pencil reconstruction and visualization of their position in the *hypogeum*: **a** “Teoria di Santi”; **b** “Madonna in trono con bambino”; **c** “S. Giorgio nell’atto di uccidere il drago”

The *hypogeum* contains three main frescos (Fig. 2) dated back to the mid-twelfth to thirteenth century AD (Fiorillo 2020): one, on the southern wall of the eastern room of the *frigidarium*, presents the Virgin seated on a throne with Jesus, S. Giacomo, S. Pietro, S. Caterina d’Alessandria and an unidentified saint (“Teoria di Santi” in Fig. 2a); the second one depicting a Madonna on the throne with a Child on the right and S. Caterina d'Alessandria on the left (“Madonna in trono con bambino” in Fig. 2b); the last one depicts S. Giorgio on horseback slaying the dragon and S. Nicola dressed in bishop’s robes (“S. Giorgio nell’atto di uccidere il drago” in Fig. 2c).

The Monumental Complex of *S. Pietro a Corte* is the most important Longobard construction in Salerno and the only archaeological find from the Roman Period in Salerno, in a truly exceptional stratigraphic, and therefore historical, succession that has marked almost all eras up to the present day. So, there is great interest in avoiding the deterioration of the frescos found in its *hypogeum*.

## Materials and methods

### Sampling

Samplings of the efflorescences and the water of the well near the *frigidarium* of the Monumental Complex of *S. Pietro a Corte* and the Rafastia and the Fusandola rivers were performed for about six months in 2018, from 15 January to 9 July, collecting water samples once a month in triplicate. The obtained three samples were combined into a single representative sample. The sampling point for both rivers was always the same and near their source.

Glass bottles (volume of ~ 250 mL) were filled with water and immediately closed and stored between 4 and 7 °C before the analysis. A total of 21 water samples were collected and analysed.

Efflorescence samples were collected by the superficial scraping of three damaged frescos (“Teoria di Santi”, “Madonna in trono con bambino”, and “S. Giorgio nell’atto di uccidere il drago”) in *S. Pietro a Corte* and one wall inside the adjacent *Palazzo Fruscione*, in order to make a comparison between them. A total of 9 efflorescence samples (6 from *S. Pietro a Corte* and 3 from *Palazzo Fruscione*) were collected and analysed. Each efflorescence sample from *S. Pietro a Corte* consisted of about 1 g of salt, obtained by mixing salts recovered from the three damaged frescos. In the same way, 1 g of salt was collected from the wall of *Palazzo Fruscione* for each of the three samples. A description of collected water and efflorescence samples is reported in Table 1, for more details regarding sampling date of all samples see Table S1.

**Table 1:** Description of collected samples based on sampling points (with their altitude and geographic coordinates) and number of samples (N)

Sampling point	Abbreviation	Altitude over the sea (m)	Geographic position	N
The Rafastia river	RR	87±1	40.687 N 14.687 E	7 (RR1–RR7)
The Fusandola river	FR	81±1	40.680 N 14.753 E	7 (FR1–FR7)
Well in the *frigidarium*	WF	4±1	40.658 N 14.755 E	7 (WF1–WF7)
Efflorescences of *S. Pietro a Corte*	EFSPC	7±1	40.658 N 14.755 E	6 (EFSPC1–EFSPC6)
Efflorescences of *Palazzo Fruscione*	EFPF	10±1	40.680 N 14.758 E	3 (EFFP1–EFFP3)

### Analytical techniques

#### Ion-chromatography analyses

Anion-exchange chromatography analyses of water and efflorescences sampled were performed using a Thermo Scientific-Dionex^TM^ Aquion^TM^ ion chromatograph equipped with a Dionex IonPac AS23 carbonate eluent anion-exchange column, with a precision of 1%. Nitrate (NO_3_^-^), sulphate (SO_4_^2-^), and chloride (Cl^-^) ion concentrations (expressed as mg/L) were obtained using calibration curves prepared employing NaNO_3_, NaCl, and Na_2_SO_4_ as standard. The precision, expressed as one standard deviation, was 1% for all the ions considered.

Prior to the ion chromatography analysis, a solution of efflorescences was prepared by dissolving 100 mg of them in 100 mL of Millipore® highly distilled water, then filtered at 0.45 μm and diluting a hundred times. All the salts used for the measurements (sodium carbonate (Na_2_CO_3_), sodium hydrogen carbonate (NaHCO_3_), sodium nitrate (NaNO_3_) and sodium sulphate (Na_2_SO_4_), and sodium chloride (NaCl)) were purchased from Sigma-Aldrich (St. Louis, MO, USA).

#### X-ray diffraction analyses

X-ray diffraction analyses on the efflorescences were carried out with Bruker D8 Advance automatic diffractometer operating with a nickel-filtered CuKα radiation, recording data in the 2θ range of 4–80° with the resolution of 0.02°.

#### Nitrogen stable isotope ratio analyses

Isotopic analyses at natural abundance were performed by using a continuous flow isotope ratio mass spectrometry (CF-IRMS), a mass spectrometric method that gives the stable isotopic composition of a sample relative to that of a reference material (Zanasi et al. 2006; Motta et al. 2009, 2020, 2021; Proto et al. 2014a; Pironti et al. 2016, 2017, 2020a, b, 2021; Ricciardi et al. 2020).

In particular, A Delta Plus V isotope ratio mass spectrometer (ThermoFinnigan, Bremen, Germany) was used to measure δ^15^N, coupled with a Flash EA 1112 elemental analyser (ThermoFinnigan) to avoid sample pre-treatment (Paul et al. 2007). Water samples were dried to obtain solids (signed as WFD1-6 samples) for IRMS measurements. The nitrogen isotope ratio was expressed in δ‰ relative to atmospheric N_2_ (R = 0.003676) according to the following equation as reported by Brand et al. (Brand et al. 2014):

1 $$ {\delta}^{15}\mathrm{N}=\frac{\left( RSA- RREF\right)}{RREF} $$

- RSA is the respective isotope ratio of a sample (number of ^15^N atoms/number of ^14^N atoms or as approximation ^15^N/^14^N).
- RREF is the isotope ratio of the relevant internationally recognized as standard (AIR).

The delta values are multiplied by 1000 and are expressed in units “per mil” (‰). δ^15^N was calculated against internationally recognized reference materials (Brand et al. 2014) as L-glutamic acid USGS 40 (δ^15^N: – 4.52 ± 0.06‰) and potassium nitrate IAEA-NO_3_ (δ^15^N: + 4.72 ± 0.13‰), purchased from the International Atomic Energy Agency (IAEA). The precision of measurement, expressed as one standard deviation, was 0.1‰.

## Results and discussion

The first part of this work regards the chemical investigations of the water of the two important streams (the Rafastia and the Fusandola) of the city of Salerno, which feed the underground aquifer of the historic centre, to discover which of them reaches the well found in the *frigidarium* of the Monumental Complex of *S. Pietro a Corte*.

To answer this question, we collected samples of water from the two rivers and from the well for about six months and analysed them by ionic chromatography. In Table 2, the results of all the determinations as average anionic concentrations are reported (see Table S1 for all analytical results). The period of sampling was quite broad (6 months); therefore, a one-way ANOVA test (Tables S3, S4, and S5 in the supporting info) was performed to evaluate the differences between the means of the different sampling groups. At the 0.05 level, the population means were not significantly different (p value close to 1).

**Table 2:** Average anionic concentrations detected at the three different sampling points with the number of samples (N) and standard deviation (σ)

Sample	Nitrate (mg/L)	N; σ; 2σ	Sulphate (mg/L)	N; σ; 2σ	Chloride (mg/L)	N; σ; 2σ
RR	67.3	7; 0.9; 1.8	48.3	7; 0.4; 0.8	23.2	7; 0.5; 1.0
FR	26.4	7; 0.8; 1.6	68.4	7; 0.5; 1.0	22.3	7; 0.6; 1.2
WF	69.2	7; 0.9; 1.8	49.3	7; 0.5; 1.0	24.1	7; 0.4; 0.8

For all the considered anions, the average values discovered for the water of the well in the *frigidarium* are close to that of the Rafastia river suggesting that this stream reaches the *hypogeum* of the Monumental Complex of *S. Pietro a Corte*.

**Fig. 3:**
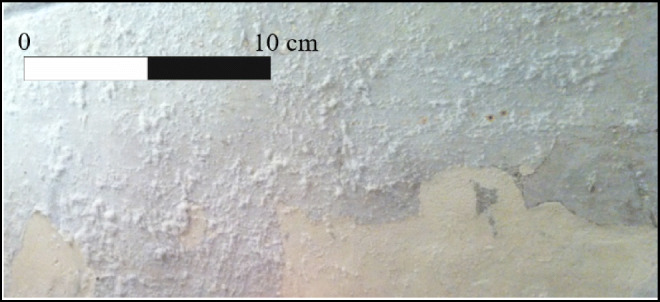
Salt efflorescences on the wall of the *hypogeum*

Considering these results, we moved to the chemical characterization of the efflorescences that occur on the wall of *hypogeum* near several frescoes, damaging them as shown in Fig. 3.

First of all, the ionic analysis of the prepared water samples of efflorescences (see Table 3 and Table S2) proved that the salt coating on the frescos is principally made of nitrate (minimum 90% w/w) with a small fraction of sulphate (less than 10 % w/w).

**Table 3:** Average nitrate content (% w/w) and nitrogen stable isotope ratio of efflorescences from *S. Pietro a Corte* (EFSPC) and *Fruscione Palace* (EFFP), and salts from the well in the *frigidarium* (WFD).

Sample	Nitrate (%)	N; σ; 2σ	δ ^15^N (‰)	N; σ; 2σ
EFSPC	95	6; 3; 6	+ 9.3	6; 0.2; 0.4
EFFP	92	3; 2; 4	+ 8.6	3; 0.2; 0.4
WFD	49	6; 1; 2	+ 2.9	6; 0.3; 0.6

**Fig. 4:**
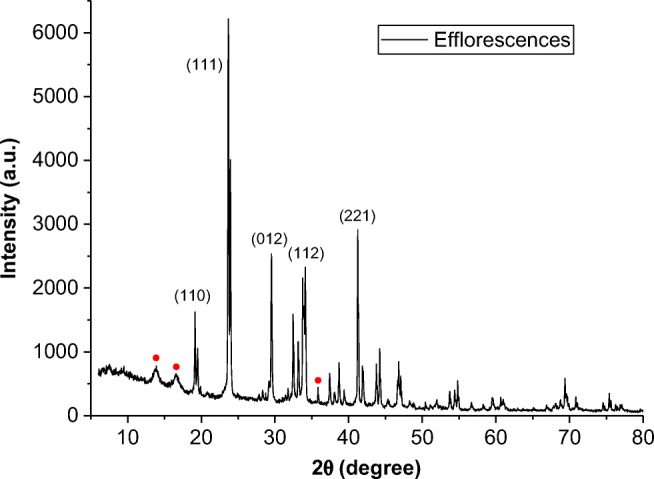
X-ray diffraction spectrum of one of the efflorescence samples (EFSPC5) with identification of the main potassium nitrate signals; red circles indicate peaks not assigned to potassium nitrate

Furthermore, X-ray diffraction spectra collected for efflorescences (see Fig. 4 for one example and Figure S3-S5 for others) have shown the typical diffraction pattern of potassium nitrate (Crystallography Open Database 2021; Yildirim et al. 2015; Sharma et al. 2018). In fact, peaks with 2*θ* (hkl) 19° (110), 19.5 (020), 23.5° (111) 24° (021), 29 (012), 32 (102), 33 (200), 34 (112), 37 (211), 38.2 (122), 38.7 (220), 39.5 (040), 41 (221), 42 (041), 43.7 (202), 44.3 (132), 46.6 (023), 54.5 (241), 60.7 (114), 66.9 (332), and 70 (153) were assigned to the orthorhombic crystal structure (α-phase) of potassium nitrate. This diffraction pattern was observed in all samples, while only a few signals present in the spectra were not assigned (red circles in Fig. 4 and Figure S4-S5), and their intensities were in accord with nitrate content. In details, sample EFSPC1 (98% of nitrate) has no additional peaks, while sample EFFP3 (90 % of nitrate) has well-distinguished peaks not assignable to potassium nitrate and spectra of sample EFSPC5 (Fig. 4) with an intermediate nitrate content (95% of nitrate) shows unassigned peaks with very low intensity.

From these results, we can assert that the efflorescences that damage frescos in *S. Pietro a Corte* are mainly made of saltpetre, so we wondered about its possible origin. In our case, a plausible source of saltpetre efflorescences is represented by the well near to *frigidarium*, in which we have detected a high concentration of nitrates (69.2 ± 0.9 mg/L).

To answer this question, we carried out δ^15^N isotopic analyses on the efflorescences and the salts recovered from the waters of the well by using isotope ratio mass spectrometry (IRMS). As we can see from the results reported in Table 3, the nitrates recovered from the water of the well have a value of nitrogen stable isotope ratio (+ 2.9 ± 0.3‰) different from that of the efflorescences present on the walls (+ 9.3 ± 0.2‰). Therefore, the deterioration of the walls caused by the efflorescences is not originated from the water of the well in the *frigidarium*.

The δ^15^N value of + 2.9‰ is generally consistent with the inorganic nitrogen fertilizers, having a typical δ^15^N value range between − 6 and + 6 ‰ (Xue et al. 2009), and this result could be explained considering that the nitrates determined in the well certainly originate from the leaching of the fertilizers used in the agricultural lands crossed by the streams (Bateman and Kelly 2007).

On the contrary, organic fertilizers such as plant composts and animal waste, have higher and more variable δ^15^N values (from + 2 to + 30‰) than inorganic fertilizers, due to their more different origins (Kendall et al. 2007). Animal waste products, in fact, are enriched in ^15^N relative to other nitrogen sources, because of volatilization of ^15^N-depleted ammonia, and subsequent oxidation of the residual waste material (containing ^15^N-enriched NH_4_^+^) into nitrate with a high δ^15^N value. Consequently, δ^15^N values of nitrate originating from animal waste are in the range of + 4 to + 25‰ (Xue et al. 2009).

Probably, the higher δ^15^N value of nitrate of the efflorescences (+ 9.3 ± 0.2‰) is related to degradation phenomena of organic substances different from those present in the well. Moreover, we also detected the formation of the same salts, with a similar isotopic delta value (8.6 ± 0.2‰), inside the ancient *Palazzo Fruscione* which is located on the north side of *S. Pietro a Corte*. Values of δ^15^N consistent with the degradation of organic substances have been previously reported in other ancient architectural sites: in the “entombment of Christ” sculpture group located in Pont-à-Mousson, France (Kloppmann et al. 2014), the nitrogen stable isotope ratio ranges from + 7.3 to + 9.5‰ suggesting an organic origin of nitrates, derived from animal waste contamination of the alluvial aquifer; the nitrate efflorescences on the sculptures of Burgos Cathedral, Spain (Gázquez et al. 2015), present a value δ^15^N of +15.4‰ related to the anthropic pollution of the groundwater caused by farming and sewage leaks.

In order to find an explanation for the formation of efflorescences in *S. Pietro a Corte*, we supported the chemical analysis with an in-depth study of the historical and geographical documents concerning the historic centre of Salerno and in particular the relationship between this Monumental Complex and the aquifer of the city. Historical sources (Amarotta 1989; Miccio 2011) have highlighted the presence of a sedimentary layer whose size and composition have varied over the years following documented flooding episodes in the city. Among the most recent catastrophes, the flood that occurred in 1954 caused extensive damage to the historic city centre as reported by several documents (Braca et al. 2007; Violante et al. 2017). Moreover, the evaluation of the variability of nitrate content is important to assess the connectivity between groundwater and surface water (Stellato et al. 2016).

Considering the case under study, the road that divides the two buildings (*S. Pietro a Corte* e *Palazzo Fruscione*) is crossed by drains of rainwater and pipes for collecting wastewater that probably not support the anthropogenic load of the neighbourhood (Curt et al. 2004; Motta et al. 2008; Proto et al. 2014b; Wigand et al. 2007; Vigliotta et al. 2010).

**Fig. 5:**
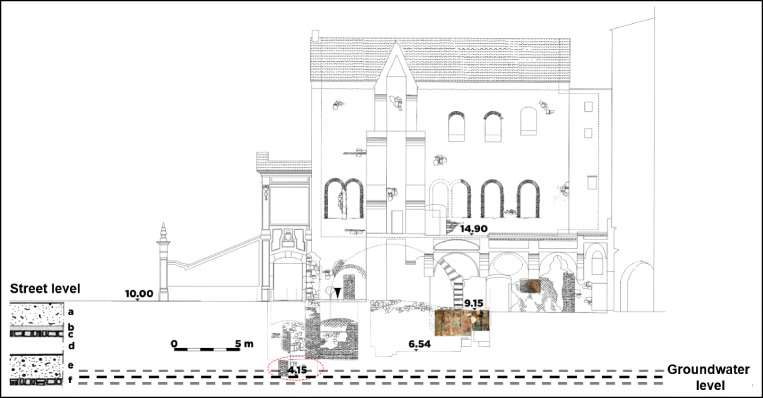
Picture of the Monumental Complex of *S. Pietro a Corte* with the stratification of sediment layer (**a** silt and sand; **b** trodden soil; **c** backfill material; **d** cobblestones from Longobard age; **e** backfill material; **f** sand and gravel) above the current street level. Dotted red circle indicates the well, while the inverted triangle (▼) indicates the altitude above sea level. Dotted black line represents the average groundwater level, whereas the grey ones indicate the variation of this level during the year.

As shown in Fig. 5, below the current street level, it is possible to recognize six different layers. Starting from the bottom, there are a layer made of sand and gravel transported by the flood dated fourth–fifth century AD (f) and consequent backfill material (e), cobblestones from Longobard age (d), further backfill material (c), a thin layer of trodden soil (b), and a final layer made of silt and sand formed as a result of another flood occurred in ninth–tenth century AD (a). Most of the components of these sediment layers are highly permeable (sand, gravel, etc.), but the layer “d” is made of impermeable material as reported in historical documents of the city (Amarotta 1989). Therefore, this layer almost always prevents the mixing of groundwater with rainwater and sewage water. As the *hypogeum* is located below the average level of the sewerage system in the historic centre of Salerno, rainwater and sewage water could permeate through the walls of the upper layers of the monumental complex, and consequently reach the *hypogeum* and cause the formation of saltpetre efflorescences on the frescos.

## Conclusions

In this paper, the nature and a plausible origin of the efflorescences that damaged several frescos in the *hypogeum* of the Monumental Complex of *S. Pietro a Corte*, in Salerno (Campania, Italy), were investigated through chemical and isotopic analyses.

Thanks to the ion-exchange chromatography analyses of water samples, we can assert that the Rafastia river feeds the ancient *frigidarium* of *S. Pietro a Corte.* X-ray diffraction and anionic chromatography allowed the determination of the chemical composition of the efflorescences: they are principally made of nitrate in the form of saltpetre. Nitrogen stable isotopic ratio analyses were employed to find out if the salts contained in the water of the well in the *frigidarium* are responsible for the formation of the efflorescences on nearby frescos. δ ^15^N measurements have shown that the nitrates recovered from the water of the well have a value of nitrogen stable isotope ratio (+ 2.9 ± 0.3‰) different from that of the efflorescences present on the walls (+ 9.3 ± 0.2‰), indicating that the two rivers are not liable for their origination. Therefore, a plausible cause of the deterioration of the Monumental Complex could be the sewage waters that permeate through the walls containing the frescos. Our hypothesis is mainly based on the chemical and isotopic analysis of the samples and the study of historical and geographical documents. A detailed investigation of the lithology and hydrology of the historic centre of Salerno and the effects of important rain events such as floods characterized by the mix of river water, groundwater, and sewage water can be an important subject of future researches.

Based on these results, restoration actions should be directed towards an improvement of the city’s sewerage system and constant maintenance of it.

## Supplementary information

ESM 1 (DOCX 1994 kb)

## Data Availability

Not applicable.
