# Supplementary material for: Investigations on historical monuments’ deterioration through chemical and isotopic analyses: an Italian case study
Source: Environ Sci Pollut Res Int. 2021 Jun 29;29(20):29409–18. doi: 10.1007/s11356-021-15103-x (PMC9001298; doi:10.1007/s11356-021-15103-x)
Supplement: Supplementary file 1 — (DOCX 1994 kb) [file 11356_2021_15103_MOESM1_ESM.docx]

**Supporting information**

**Chemical characterization and stable isotope composition of efflorescensces developed on frescos of an Italian Monumental Complex**

Maria Ricciardi^1^, Concetta Pironti^1^, Oriana Motta^1*^, Rosa Fiorillo^2^, Federica Camin^3,4^, Antonio Faggiano^5^, Antonio Proto^5^

*^1^Department of Medicine Surgery and Dentistry, University of Salerno, via S. Allende, 84081 Baronissi (SA), Italy*

*^2^Department of Cultural Heritage, University of Salerno, via Giovanni Paolo II 132, 84084 Fisciano (SA), Italy*

*^3^Food Quality and Nutrition Department Research and Innovation Centre, Fondazione Edmund Mach (FEM), Via E. Mach 1, 38010 San Michele all'Adige (TN), Italy*

*^4^Center Agriculture Food Environment (C3A), University of Trento, via Mach 1, 38010 San Michele all'Adige (TN)*

*^5^Department of Chemistry and Biology, University of Salerno, via Giovanni Paolo II 132, 84084 Fisciano (SA), Italy*

***Corresponding Author: Prof. Oriana Motta, University of Salerno via S. Allende, 84081 Baronissi (SA), Italy tel +39 89963083, e-mail: omotta@unisa.it

**Summary**

[**Images of Monumental complex structure and well in the frigidarium** 3](#_Toc72820135)

[**Figure S1**. (a) Section of Monumental Structure; (b) Magnification of Hypogeum: frigidarium (with a circle to indicate the well) and paleochristian church map. 3](#_Toc72820136)

[**Figure S2**. Picture of the well close to the frigidarium: front view (a) and top view (b). 3](#_Toc72820137)

[**Chemical and isotopic characterizations of samples with corresponding sampling date** 4](#_Toc72820138)

[**Table S1**. Anionic concentrations detected at the three different sampling points with sampling date. 4](#_Toc72820139)

[**Table S2**. Nitrate and sulphate content (% w/w) and nitrate stable isotope ratio for efflorescence samples and dried samples of water from the frigidarium. 5](#_Toc72820140)

[**X-ray diffraction spectra** 6](#_Toc72820141)

[**Figure S3**. X-ray diffraction spectrum of efflorescence sample EFSPC1 from *San Pietro a Corte* with identification of the main potassium nitrate signals. 6](#_Toc72820142)

[**Figure S4**. X-ray diffraction spectrum of dried water sample from the well of *San Pietro a Corte* with identification of the main potassium nitrate signals; red circles indicate peaks not assigned to potassium nitrate. 6](#_Toc72820143)

[**Figure S5**. X-ray diffraction spectrum of efflorescence sample EFFP2 from *Palazzo Fruscione* with identification of the main potassium nitrate signals; red circles indicate peaks not assigned to potassium nitrate. 7](#_Toc72820144)

[**One-way ANOVA** 7](#_Toc72820145)

[**Table S3**. ANOVA test for nitrates. 7](#_Toc72820146)

[**Table S4**. ANOVA test for sulphates. 7](#_Toc72820147)

[**Table S5**. ANOVA test for chloride. 8](#_Toc72820148)

# **Images of Monumental complex structure and well in the frigidarium**


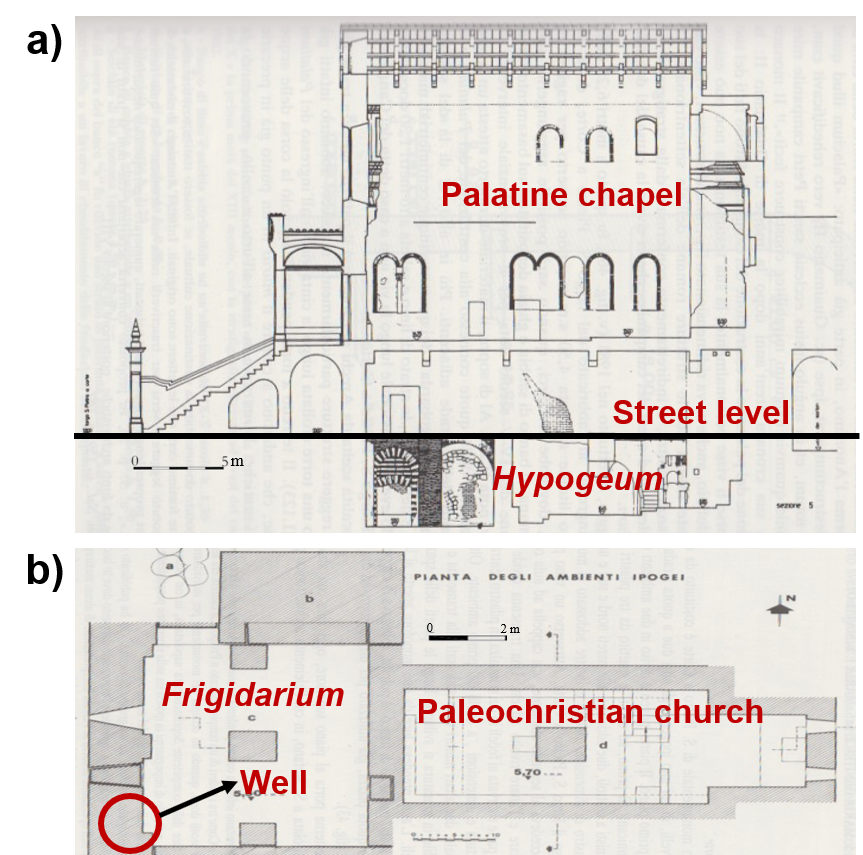


## **Figure S1**. (a) Section of Monumental Structure; (b) Magnification of Hypogeum: frigidarium (with a circle to indicate the well) and paleochristian church map.


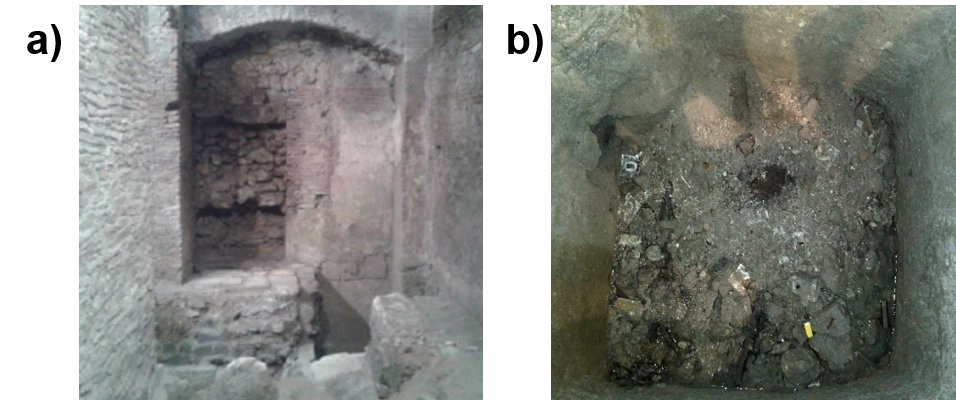


## **Figure S2**. Picture of the well close to the frigidarium: front view (a) and top view (b).

# **Chemical and isotopic characterizations of samples with corresponding sampling date**

## **Table S1**. Anionic concentrations detected at the three different sampling points with sampling date.

| **Sampling**  **date (d, m, y)** | **Sample** | **Nitrate**  **(mg/L)** | **Sulphate**  **(mg/L)** | **Chloride (mg/L)** |
| --- | --- | --- | --- | --- |
| 15/01/2018 | RR1 | 66.8 | 47.7 | 22.8 |
|  | FR1 | 26.0 | 68.0 | 21.9 |
|  | WF1 | 68.6 | 48.7 | 23.7 |
| 12/02/2018 | RR2 | 68.0 | 48.9 | 23.7 |
|  | FR2 | 27.1 | 69.2 | 23.0 |
|  | WF2 | 69.8 | 49.9 | 23.8 |
| 19/03/2018 | RR3 | 67.0 | 48.4 | 23.6 |
|  | FR3 | 26.1 | 68.3 | 22.0 |
|  | WF3 | 68.5 | 49.5 | 24.0 |
| 16/04/2018 | RR4 | 66.6 | 47.8 | 22.5 |
|  | FR4 | 25.3 | 67.9 | 21.7 |
|  | WF4 | 68.1 | 48.7 | 23.6 |
| 14/05/2018 | RR5 | 69.0 | 48.6 | 23.4 |
|  | FR5 | 27.6 | 68.6 | 22.2 |
|  | WF5 | 70.5 | 49.3 | 24.3 |
| 11/06/2018 | RR6 | 67.3 | 48.6 | 23.7 |
|  | FR6 | 26.6 | 68.7 | 21.8 |
|  | WF6 | 69.8 | 49.8 | 24.6 |
| 09/07/2018 | RR7 | 66.7 | 48.2 | 23.0 |
|  | FR7 | 25.8 | 68.3 | 23.2 |
|  | WF7 | 69.1 | 49.1 | 24.5 |

## **Table S2**. Nitrate and sulphate content (% w/w) and nitrate stable isotope ratio for efflorescence samples and dried samples of water from the frigidarium.

| **Sampling**  **date (d, m, y)** | **Sample** | **Nitrate**  **(%)** | **Sulphate**  **(%)** | **δ ^15^N**  **(‰)** |
| --- | --- | --- | --- | --- |
| 15/01/2018 | EFSPC1 | 98 | 2 | 9.1 |
|  | EFFP1 | 94 | 6 | 8.4 |
|  | WFD1 | 49 | 34 | 3.0 |
| 12/02/2018 | EFSPC2 | 97 | 3 | 9.4 |
|  | WFD2 | 49 | 35 | 2.6 |
| 19/03/2018 | EFSPC3 | 96 | 4 | 9.5 |
|  | EFFP2 | 93 | 7 | 8.9 |
|  | WFD3 | 48 | 35 | 3.4 |
| 14/05/2018 | EFSPC4 | 90 | 10 | 9.3 |
|  | WFD4 | 49 | 34 | 2.5 |
| 11/06/2018 | EFSPC5 | 95 | 5 | 9.5 |
|  | WFD5 | 48 | 35 | 2.9 |
| 09/07/2018 | EFSPC6 | 94 | 6 | 9.2 |
|  | EFFP3 | 90 | 10 | 8.6 |
|  | WFD6 | 48 | 34 | 2.7 |

# **X-ray diffraction spectra**

## **Figure S3**. X-ray diffraction spectrum of efflorescence sample EFSPC1 from *San Pietro a Corte* with identification of the main potassium nitrate signals.

## **Figure S4**. X-ray diffraction spectrum of dried water sample from the well of *San Pietro a Corte* with identification of the main potassium nitrate signals; red circles indicate peaks not assigned to potassium nitrate.

## **Figure S5**. X-ray diffraction spectrum of efflorescence sample EFFP2 from *Palazzo Fruscione* with identification of the main potassium nitrate signals; red circles indicate peaks not assigned to potassium nitrate.

# **One-way ANOVA**

Independent variable: sampling date; dependent variable: anionic concentration (nitrate, sulphate and chloride). Tables S3, S4 and S5 report the degrees of freedom (Df, calculated as N-1 where N is the number of sampling days, the residuals are calculated as the total samples minus the sampling dates), the sum of the squares, the mean of the squares, the F value for the comparison of the variances (ratio between Mean Sq sampling dates and residuals), and the p value (Pr(>F)) to accept or reject the null hypothesis.

|  | **Df** | **Sum Sq** | **Mean Sq** | **F value** | **Pr(>F)** |
| --- | --- | --- | --- | --- | --- |
| **Sampling dates** | 6 | 12 | 2.0 | 0.003 | 1 |
| **Residuals** | 14 | 8211 | 586.5 |  |  |

## **Table S3**. ANOVA test for nitrates.

## **Table S4**. ANOVA test for sulphates.

|  | **Df** | **Sum Sq** | **Mean Sq** | **F value** | **Pr(>F)** |
| --- | --- | --- | --- | --- | --- |
| **Sampling dates** | 6 | 3.6 | 0.6 | 0.005 | 1 |
| **Residuals** | 14 | 1801.5 | 128.7 |  |  |

## **Table S5**. ANOVA test for chloride.

|  | **Df** | **Sum Sq** | **Mean Sq** | **F value** | **Pr(>F)** |
| --- | --- | --- | --- | --- | --- |
| **Sampling dates** | 6 | 2.345 | 0.3908 | 0.4 | 0.867 |
| **Residuals** | 14 | 13.693 | 0.9781 |  |  |
